# Research progress of 3D printed poly (ether ether ketone) in the reconstruction of craniomaxillofacial bone defects

**DOI:** 10.3389/fbioe.2023.1259696

**Published:** 2023-08-16

**Authors:** Qiao Su, Yixin Qiao, Yile Xiao, Shuhao Yang, Haoming Wu, Jianan Li, Xinlong He, Xulin Hu, Hui Yang, Xin Yong

**Affiliations:** ^1^ Key Laboratory of Birth Defects and Related Diseases of Women and Children, Department of Paediatrics, West China Second University Hospital, State Key Laboratory of Biotherapy and Collaborative Innovation Center of Biotherapy, Sichuan University, Chengdu, Sichuan, China; ^2^ State Key Laboratory of Oral Diseases and National Clinical Research Center for Oral Diseases and West China Hospital of Stomatology, Sichuan University, Chengdu, Sichuan, China; ^3^ West China School of Stomatology, Sichuan University, Chengdu, Sichuan, China; ^4^ Department of Otolaryngology-Head and Neck Surgery, West China Hospital, Sichuan University, Chengdu, Sichuan, China; ^5^ Beijing Shijitan Hospital, Capital Medical University, Beijing, China; ^6^ Clinical Medical College and Affiliated Hospital of Chengdu University, Chengdu, Sichuan, China; ^7^ State Key Laboratory of Biotherapy, State Key Laboratory and Collaborative Innovation Center of Biotherapy, West China Hospital, Sichuan University, Chengdu, Sichuan, China

**Keywords:** polyetheretherketone, 3D printing, modification technology, craniomaxillofacial bone defect, bone reconstruction

## Abstract

The clinical challenge of bone defects in the craniomaxillofacial region, which can lead to significant physiological dysfunction and psychological distress, persists due to the complex and unique anatomy of craniomaxillofacial bones. These critical-sized defects require the use of bone grafts or substitutes for effective reconstruction. However, current biomaterials and methods have specific limitations in meeting the clinical demands for structural reinforcement, mechanical support, exceptional biological performance, and aesthetically pleasing reconstruction of the facial structure. These drawbacks have led to a growing need for novel materials and technologies. The growing development of 3D printing can offer significant advantages to address these issues, as demonstrated by the fabrication of patient-specific bioactive constructs with controlled structural design for complex bone defects in medical applications using this technology. Poly (ether ether ketone) (PEEK), among a number of materials used, is gaining recognition as a feasible substitute for a customized structure that closely resembles natural bone. It has proven to be an excellent, conformable, and 3D-printable material with the potential to replace traditional autografts and titanium implants. However, its biological inertness poses certain limitations. Therefore, this review summarizes the distinctive features of craniomaxillofacial bones and current methods for bone reconstruction, and then focuses on the increasingly applied 3D printed PEEK constructs in this field and an update on the advanced modifications for improved mechanical properties, biological performance, and antibacterial capacity. Exploring the potential of 3D printed PEEK is expected to lead to more cost-effective, biocompatible, and personalized treatment of craniomaxillofacial bone defects in clinical applications.

## 1 Introduction

**FIGURE 1:**
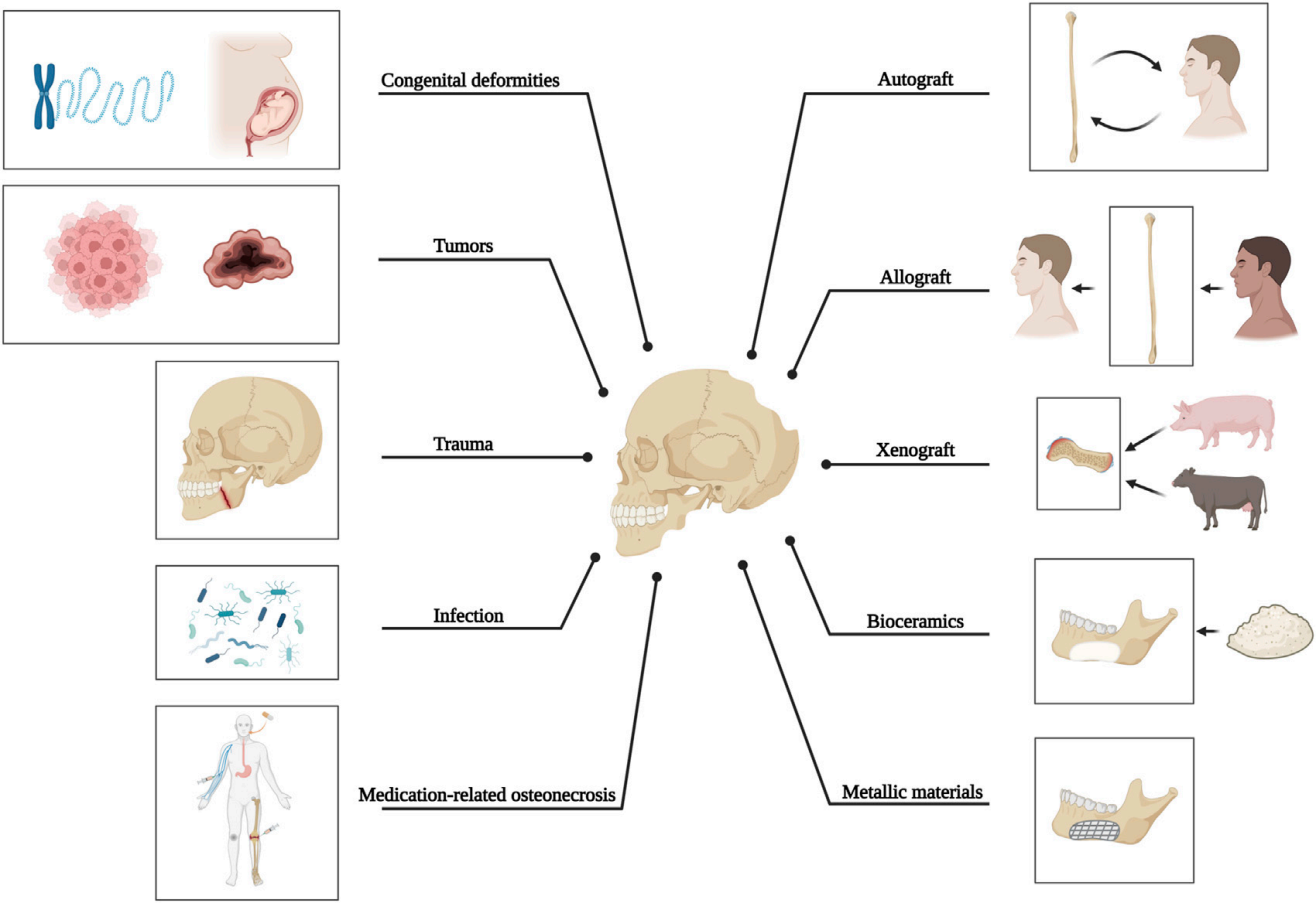
Causative factors of craniomaxillofacial bone defects and current methods applied for bone reconstruction.

The anatomical complexity of craniomaxillofacial bone defects resulting from tumors, trauma, infection, or congenital deformities often presents significant clinical challenges. In cases where bone defects are too extensive for natural healing processes, autologous bone grafting is the primary option due to its osteogenic, osteoinductive, and osteoconductive capacity, although the utilization of this technique is limited by constraints such as donor site mismatch, bone graft resorption, and the occurrence of infection. Therefore, metals such as titanium and its alloys are highly preferred synthetic materials due to their excellent mechanical robustness, corrosion resistance, and compatibility with living organisms. However, there are several significant disadvantages, one of which is stress-shielding at the junction of titanium and bone. The opacity of metals in images and the release of detrimental metal ions also hinder and impede their use (Figure 1).

Because of these limitations, researchers are investigating polymers as potential substitutes for bone reconstruction, with poly (ether ether ketone) (PEEK) being extensively studied as a viable alternative to commonly used materials. As a high-performance thermoplastic polymer with a polyaromatic, semicrystalline nature, PEEK exhibits remarkable mechanical characteristics similar to human bone, excellent chemical resistance, and favorable biocompatibility. And compared to zirconia and metal alloys, PEEK does not induce metal allergy and does not cause artifacts during routine imaging studies, making its application highly advantageous (Luo et al., 2023).

In addition to the development of novel materials research, additive manufacturing (AM) or 3D printing technology, which builds objects layer by layer until they are complete, offers several advantages for bone repair in terms of appearance and function, as well as the occurrence of complications, and surpasses conventional technology with unique features such as rapid prototyping, high accuracy, and minimal error rate (Derby, 2012; Feng et al., 2020; Fonseca et al., 2020; Murphy et al., 2020; Zhu et al., 2020; Mian et al., 2022). Among the various materials used in 3D printing, PEEK is considered to be excellent, customizable, and 3D-printable to replace traditional freehand-molded autografts and titanium implants to repair various forms of bone defects with identical shape and design, which is especially beneficial for maintaining the original anatomical contour and not impairing physiological functions such as mastication and joint movement in the context of craniomaxillofacial bone reconstruction (Li et al., 2022a). In recent years, the fabrication of PEEK constructs using selective laser sintering (SLS) has gained significant popularity due to the flexibility of available materials and design complexity (Deng et al., 2018; Zhao et al., 2018; Basgul et al., 2021). Fused deposition modeling (FDM) has also experienced significant growth as another printing technique for producing PEEK and its composites with affordability, simplified operation (using filaments instead of powders), and decreased likelihood of material pollution or degradation (Punchak et al., 2017).

Although PEEK material has certain advantages, its lack of bioactivity hinders the wider use of 3D printed PEEK constructs in the reconstruction of bone defects. The growing need for improved treatment efficacy and patient wellbeing requires the development of advanced constructs that can achieve rapid bone integration and provide additional beneficial therapeutic features. Consequently, the modification of 3D printed PEEK constructs has gradually evolved from providing sufficient mechanical support and improving their bioactivity to, more recently, combating bacterial infection and inflammation.

This review presents the essential requirements for craniomaxillofacial bone reconstruction and commonly used methods, and specifically summarizes the latest studies and modified approaches regarding 3D printed PEEK constructs, thus encouraging their further advancement and clinical applications in the reconstruction of complex craniomaxillofacial bone defects.

## 2 Performance demands and current methods for bone reconstruction

### 2.1 Performance demands

From a histological perspective, the structure of natural bone consists of an outer layer of cortical bone and an inner layer of cancellous bone (Liu et al., 2016). Its main mechanical properties are provided by the cortical bone, which is highly compact with less than 10% porosity. For another, 80% of bone remodeling activities occur in the cancellous bone, which consists of the plate- or rod-like structures with a porosity of 50%–90% (Bose et al., 2013; Langdahl et al., 2016; Tertuliano and Greer, 2016; Hu et al., 2023a). In particular, the mandible, which is critical in craniofacial functions, appearance, and speech, undergoes development through intramembranous ossification. As a result, it comprises durable cortical plates and a bone marrow cavity abundant in cells. Inorganic components like hydroxyapatite (HAP), associated with hardness, and organic components like collagen, associated with elasticity, comprise most of the mandible (Matsui et al., 2021).

Even though the bone is widely recognized for its ability to self-heal, critical-sized bone defects cannot be restored entirely without external interventions. Cranial defects can occur from decompressive craniectomy due to infection, injury, or removal of intracranial tumors that invade the bone. Cranioplasty, a surgical procedure for reconstructing the skull, is often necessary to restore cranial defects’ anatomy, aesthetics, and function. For another, to address mandibular bone defects, which refer to abnormalities in the lower jaw with or without injury to the facial bones and their accessories (Akinbami, 2016), resulting from congenital malformations (Forbes, 2010), tumors (Thariat et al., 2012), trauma (Berg et al., 2014), inflammation (Zhou et al., 2018), or medication-related osteonecrosis (Zhu et al., 2022), it is also critical to achieve anatomical (Kang et al., 2021), aesthetic (Batstone, 2018), and functional restoration (Kakarala et al., 2018), while withstanding the challenging conditions posed by oral microflora, lifelong stress during mastication, and continuous force exerted by adjacent tissues (Park et al., 2022). Therefore, it can be concluded that in order to create optimal reparative constructs for bone tissue reconstruction in the craniomaxillofacial area, the structure of these constructs should simulate natural bone in terms of the mechanical, bioactive, and other functional properties (i.e., structural durability, biological performance, and protection against bacterial infection).

To begin with, the durability of reparative materials relies heavily on mechanical properties, particularly the suitable elastic modulus, which is crucial for preventing stress shielding at the healing site (i.e., bone destruction and bone resorption) (Papathanasiou et al., 2020). Therefore, it is essential to establish specific mechanical requirements for the material to achieve better repair outcomes, including appropriate initial mechanical strength, suitable elastic modulus, and a favorable strength degradation rate aligned with those of natural bone, as shown in Table 1. Both trabecular and cortical bones of the maxilla and mandible exhibit anisotropy, meaning their mechanical characteristics differ based on direction. Furthermore, the mandible demonstrates superior physical attributes to the maxilla regarding both the outer and inner layers of bone, and the mechanical properties of these two layers also vary (Dorado et al., 2022).

**TABLE 1:** Mechanical properties of craniomaxillofacial bones, titanium, and PEEK (Misch et al., 1999; O’Mahony et al., 2000; Schwartz-Dabney and Dechow, 2002; 2003; Lettry et al., 2003; Seong et al., 2009; Han et al., 2019; Feng et al., 2020; Mian et al., 2022; Luo et al., 2023).

Materials	Density (g/cm^3^)	Martens hardness (N/mm^2^)	Compressive strength (MPa)	Tensile strength (MPa)	Elastic modulus (GPa)
Cortical bone	1.92			104–121	6–30
Trabecular bone	0.05–0.3		2–70		0.01–3
Maxillary bone	0.67				edentulous maxilla: 14.5 (anterior section), 15.3 (posterior section)
Mandibular cortical bone	1.85–2.0				5–15 (premolar and molar sections)
					E_1_ = 12.5, E_2_ = 17.9, E_3_ = 26.6 (edentulous mandibles), E_1_ = 12.7, E_2_ = 17.9, E_3_ = 22.8 (dentate mandibles)
					edentulous mandible: 16.8 (anterior section), 19.7 (posterior section)
MANDIBULAR TRABECULAR BONE	1.14		0.22–10.44		0.0249–0.24 (with cortical plates), 0.0035–0.1256 (without cortical plates)
					edentulous mandible: 0.91 (mesio-distal), 0.51 (bucco-lingual), 0.11 (infero-superior)
					edentulous mandible: 16.8 (anterior section), 19.7 (posterior section)
Dentin	3.3	468.2 ± 30.77		104	12–18.6
Enamel	2.6–3	2263.6 ± 405.16		47.5	40–83
Titanium	4.5	300–400		954–976	102–110
PEEK	1.32	189.55 ± 16.89		87.53–100	3–4

Favorable biological performance, such as biocompatibility and osteogenic effects, is as important as mechanical strength for biomedical applications. When examining, the pore size and porosity of constructs are critical, as research has demonstrated that nanoporous structure can enhance adhesion, spread, and osteogenic differentiation of cells, and microporous structure can enhance the transportation of nutrients and functional components within constructs, improving cell ingrowth, vascularization, and waste elimination (Bose et al., 2013; Gao et al., 2022). The pore size has a significant impact on the formation and arrangement of extracellular matrix (ECM), as the poly (D, L-lactic acid) (PDLLA) scaffolds with pores measuring 325 and 420 μm resulted in well-structured collagen I network (Stoppato et al., 2013). Conversely, scaffolds with smaller pores measuring 275 μm hindered the growth and differentiation of osteoblasts, as well as their ability to produce functional ECM. Furthermore, suitable porosity is advantageous for bone tissue regeneration *in vivo*, as the poly-ɛ-caprolactone (PCL) scaffolds with well-structured design and high permeability facilitated superior bone growth compared to the low-permeable scaffolds (Mitsak et al., 2011). The ability of these high-permeable scaffolds to allow for better bone tissue penetration and blood vessel infiltration led to enhanced mechanical performance during the compression test after being implanted in immune-compromised mice for 8 weeks. Pore parameters also influence the biodegradation kinetics of porous scaffolds, as the increased surface area per unit volume with increasing porosity plays a role (Bose et al., 2013).

To achieve better outcomes, carefully designing and balancing the pore size and porosity of constructs is essential, especially considering that tiny pores can hinder cell proliferation and limit vascularization and nutrient delivery, while large ones affect load-bearing capacity and negatively impact fatigue lifetime. To begin with, the pore size of the natural bone Harvard system is about 100–200 μm (Karageorgiou and Kaplan, 2005). The cortical bone is highly compact with less than 10% porosity, while the cancellous bone consists of plate- or rod-like structures with a porosity of 50%–90%. Particularly, the cortical porosity of the mandibular condyle, with an average of 3.5%, showed no significant variation across cortical regions, while in trabecular bone, with an average porosity of 79.3%, a notable inverse relationship existed between the surface area of the trabeculae and mineralization level (Renders et al., 2007). Therefore, suitable pore sizes, i.e., macropores from 100 to 500 μm and micropores less than 10 μm and high porosity design ranging from 50% to 90% are typically considered favorable for improved cell and tissue growth. Previous research also indicated that maintaining a 40%–70% porosity level in scaffolds is ideal for promoting proliferation, attachment, differentiation of bone cells, vascularization, and nutrient exchange (Feng et al., 2020).

The properties of the construct surface, like surface morphology (crystalline and amorphous domains), surface topography (roughness), surface affinity, surface electrical charge, and chemical composition, have potential effects on the hydrophilic nature to influence the interactions between cells and constructs for bone tissue ingrowth (Bose et al., 2013; Wu et al., 2022).

Bacterial contamination poses a significant limitation to clinical treatments of bone defects (Zhang et al., 2023), as Bakhshandeh et al. (2017) reported that the incidence of infection in orthopedic surgery ranges from 1% to 5%, with a significant exponential rise for individuals with the compromised immune system, and the likelihood of infection following revision surgery can escalate to as much as 5%–40%. When it comes to mandibular continuity defects, reconstruction plates show an infection rate between 7% and 13% (Bede et al., 2019). Mandibular distraction osteogenesis for bone regeneration also carries a 12.2% infection risk associated with the distraction device (Nørholt et al., 2011). In particular, the oral cavity is discovered to house over 700 distinct bacterial species or phylotypes, and oral fluid-borne microorganisms tend to stick together and create biofilms (Burghardt et al., 2015). These, combined with the irritation caused by food particles, may facilitate the development of implant-associated infections (IAIs) and significant loss of alveolar bone (Irie et al., 2014; Chang et al., 2021).

These findings indicate that during bone defect repair, the prevention of bacterial infection is an urgent issue. However, addressing infection is still a great challenge due to the bacteria biofilms’ strong antibiotic resistance (Wang et al., 2022a), where conventional approaches involving surgical debridement, mandibular decortication, or resections combined with prolonged systemic antibiotics are necessary to eliminate osteonecrosis or tumor of the mandible and to prevent the reoccurrence of infection (Marschall et al., 2019). Therefore, maintaining a consistent and sufficient level of antibiotic agents *in vivo* that surpasses the value of minimum inhibitory concentration (MIC) is highly encouraged to meet clinical demands. The design and fabrication of constructs with antibacterial capacity are expected to offer a practical solution to drug resistance and limited antibacterial time while stimulating bone reconstruction (Wang et al., 2017; Zhang et al., 2022).

### 2.2 PEEK as construct material for bone reconstruction

Different types of grafts, including autologous, allogenic, xenogeneic, and synthetic materials, have been utilized in maxillofacial reconstruction, each with its own set of advantages and disadvantages as outlined in Table 2. Currently, the autologous osteocutaneous vascularized free flap (OCFF) is considered the best choice for composite mandible reconstruction, and in terms of skull repair, titanium mesh, particularly the digital premolded variety, is the most commonly employed material in clinical practice (Punchak et al., 2017; Järvinen et al., 2019; Kim et al., 2020; Zhang et al., 2020; Li et al., 2022b; Guo et al., 2023). Besides, autologous cranial bone is an ideal choice due to its excellent biocompatibility and low cost, with a significantly lower complication rate than titanium mesh, although it is limited by unfavorable bone resorption (Schwarz et al., 2016).

**TABLE 2:** Advantages and disadvantages of materials commonly used in craniomaxillofacial bone reconstruction (Jeong et al., 2022; Park et al., 2022; Guo et al., 2023).

Materials	Advantages	Disadvantages
Autograft	a. Excellent biocompatibility with osteoinductive, osteoconductive, and osteogenic ability, angiogenesis, and low risk of immune rejection b. ideal structural, physiological, and anatomical properties	a. Resource scarcity b. Additional surgery and following donor site morbidity c. Limited personalization unless with a clinically experienced operator d. Time-consuming with longer operation time and healing period e. Unstable long-term results due to unpredictable bone resorption
Allograft	a. Established ossuary b. Long-term preservation c. Osteoinductive and osteoconductive ability	a. Disease spread b. immune rejection c. Hematoma and infection d. Lack the osteogenic ability of autografts
Xenograft	a. Osteoinductive and osteoconductive ability b. Abundant resource supply	a. immune rejection b. Lack the osteogenic ability of autografts
Bioceramics	a. Aesthetic properties b. Robustness	a. Low strength b. High brittleness
Metallic materials	a. Excellent mechanical strength b. Corrosion resistance c. Utility d. Shaped to match the intricate structure e. Excellent biocompatibility	a. Material breakdown and exposure entailing infection b. Metal ions induced gum discoloration and allergic reactions c. High elastic modulus induced stress shielding related to osteoporosis or implant failure over time d. Deformative change of titanium mesh during scar contracture and adjuvant radiation e. Restricted oral rehabilitation of reinforced titanium plates due to a lack of hard tissue support

All these issues motivate the improvement and development of novel materials, among which PEEK, a polyaromatic, semicrystalline, and thermoplastic polymer, is being increasingly recognized as a viable substitute for conventional repair materials, because of its ease of processing, high-temperature stability, exceptional biomechanical properties that resemble natural bone, chemical stability, color stability, and favorable biocompatibility (Papathanasiou et al., 2020; He et al., 2021; Chen et al., 2022). With lower atomic numbers, it results in fewer streaks and halo artifacts in images, making the following-ups after surgery easier. In addition to its pleasant low heat conductivity, PEEK does not induce metal allergy, which significantly benefits its use in oral and maxillofacial surgery (Rauch et al., 2020; Lommen et al., 2022; Micheletti et al., 2022).

PEEK possesses rigidity arising from the benzene ring in the molecular chain, and its adequate toughness, provided by the ether bonds, enables it to exhibit high resistance to cyclic stress (He et al., 2021). In contrast to conventional metal and ceramic materials, PEEK demonstrates remarkable lightness with an elastic modulus (3–4 GPa) relatively comparable to the cortical bone in humans (6–30 GPa) (Skinner, 1988; Luo et al., 2023), not only providing a damping effect but reducing stress shielding (Papathanasiou et al., 2020), as supported by the finding that the porous PEEK, when compared to titanium, enhanced load distribution with adjacent bone according to finite element modeling (Chen et al., 2020a). Similarly, the combination of the PEEK implant created through FDM and the free vascularized fibula graft was reported, through the finite element method, to offer exceptional safety and stability for achieving functional and aesthetic restoration of the mandible defects (Kang et al., 2021).

There is no reported notable disparity in complications between autologous cranial bone and PEEK during cranioplasty, and PEEK also does not exhibit any potential bone resorption when compared to autologous bone (Jonkergouw et al., 2016). Moreover, compared to titanium, osteoblasts and gingival fibroblasts on PEEK showed increased cellular adhesion, viability, and proliferation, indicating better cytocompatibility of PEEK (da Cruz et al., 2019).

However, the stiffness of PEEK may fail to withstand load-bearing stress, resulting in a high risk of fracture in medical applications. And its biological inertness, with a hydrophobic surface and low surface energy, makes it difficult to generate favorable cellular responses and strong and long-lasting interactions with adjacent bone tissue, resulting in limited biological effects in the field of bone regeneration. IAIs are also a challenging issue due to the bioinert nature of PEEK (Oladapo et al., 2021; Wang et al., 2022b; Luo et al., 2023).

## 3 3D printed PEEK for bone reconstruction

### 3.1 Advantages of 3D printing

In general, commonly used methods for fabricating PEEK constructs are vacuum pressing, computer-aided design/computer-aided manufacturing milling (CAD/CAM), injection molding, and thermal compression molding (Deng et al., 2015; Muhsin et al., 2019; Li et al., 2022c). However, addressing various mechanical results caused by uncontrollable factors during the molding procedure is crucial for the widespread utilization of PEEK. AM or 3D printing technology has emerged as a potential solution to this problem, offering unique benefits by constructing objects layer-by-layer via extrusion, sintering, photocuring, melting, or jetting (Matthews et al., 2017).

By providing immense flexibility in the production of intricate, irregular, and customized constructs, 3D printing brings a bright future for bone reconstruction. To be specific, the bones of different people are different in structure, morphology, etc., which necessitates the customization of artificial bone substitutes. 3D printing offers benefits in the rapid manufacture of intricate and personalized structures like ribs, mandibles, skulls, and scapulae, which ideally meet clinical requirements (Sun et al., 2022). For instance, the patient-specific cranial implants, the point-of-care (POC) PEEK constructs made using 3D printing technology, demonstrated excellent dimensional precision and consistency, displaying morphological resemblance that is clinically satisfactory in both fitting and contour continuity (Sharma et al., 2021).

The utilization of 3D printed constructs enables the superior regulation of shape, composition, pore parameters, surface characteristics, and mechanical properties compared to conventional production techniques, facilitating the creation of optimal conditions for cellular migration and proliferation, promoting the regeneration of bone tissue, and catering to the unique requirements of each patient. Although various techniques, like gas foaming, solvent casting or particulate leaching, freeze drying, thermally induced phase separation, foam-gel, and electrospinning have been employed to create bone scaffolds with porous structure, it is recommended to precisely design and manufacture constructs with customized porosity, pore size, pore shape, and interconnectivity structure for specific defects with advanced 3D printing technology (Bose et al., 2013; Lau et al., 2020). 3D printing technology enables the fabrication and modification of the framework to produce constructs with customizable characteristics by adjusting the printing techniques and printing parameters to fulfill the demands of clinical use (Feng et al., 2020).

3D printing also allows for the creation of constructs that imitate the complex structure and composition of natural bone, which can not only offer nourishment for cells and realize the delivery of bioactive factors but also contain tube-like structures to facilitate vascularization, as the hierarchical Haversian bone-mimicking scaffolds as a multicellular delivery system, produced through digital laser processing (DLP), demonstrated significantly enhanced osteogenic and angiogenic outcomes both *in vitro* and *in vivo* (Zhang et al., 2020; Guo et al., 2023).

Finally, 3D printing allows the incorporation of various elements such as bioceramics, polymers, antibacterial agents, stem cells, and bioactive factors into the created anatomically consistent constructs, combining their advantages for better osteogenesis and angiogenesis, and the treatment of infection and cancer to positively affect bone regeneration (Shukla and Maiti, 2022; Gui et al., 2023; Tripathi et al., 2023).

### 3.2 3D printed PEEK and its properties

**FIGURE 2:**
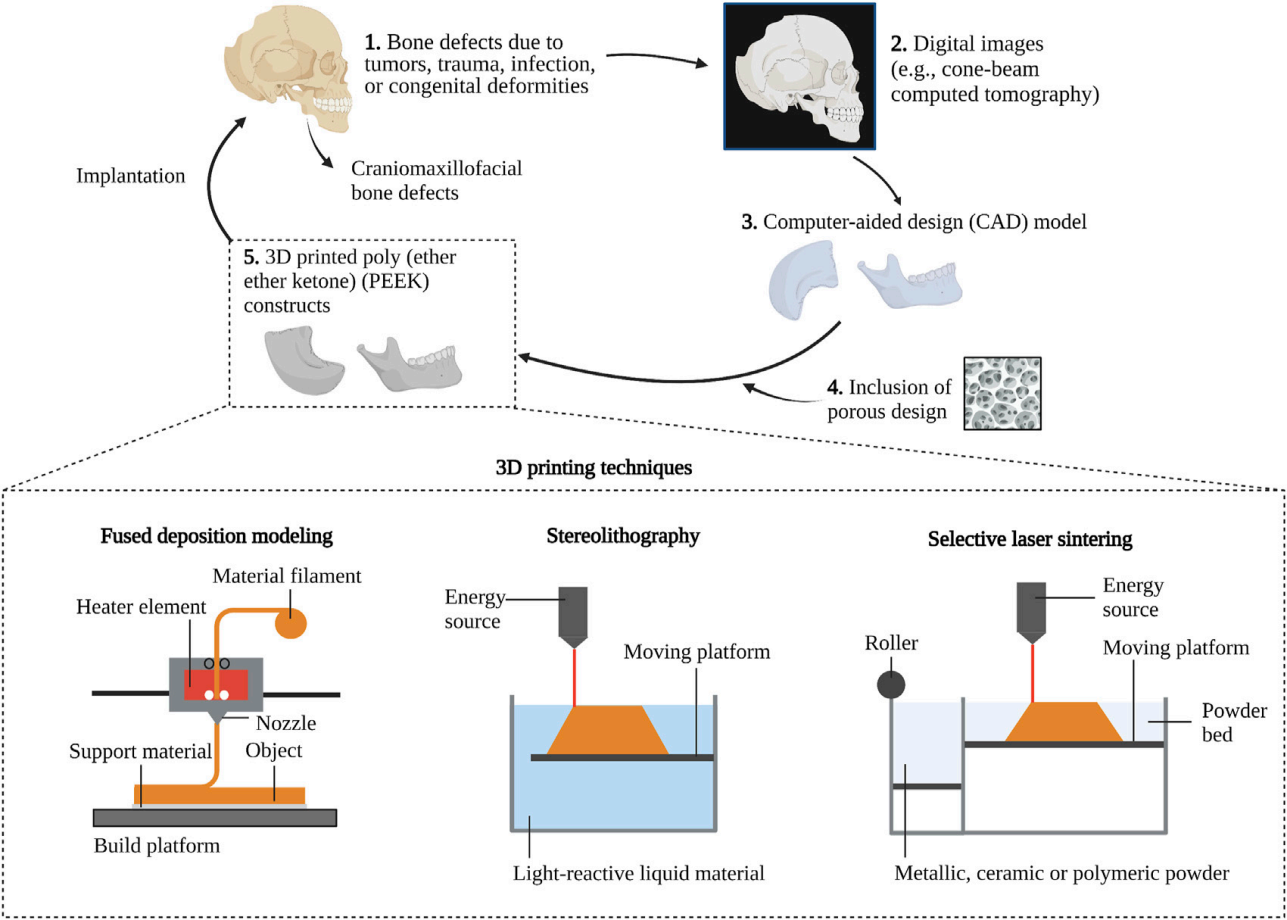
3D printing techniques for PEEK constructs in craniomaxillofacial bone reconstruction.

The current AM techniques are subjected to seven groups according to the ASTM Technical Committee, which include: 1) powder bed fusion (PBF), 2) material extrusion, 3) vat photopolymerization, 4) binder jetting, 5) material jetting, 6) directed energy deposition, and 7) sheet lamination (Chen et al., 2022). AM utilizes various raw materials, such as liquid photopolymers, powders, and filaments. The 3D printing technology for PEEK is represented by stereolithography (SLA), selective laser sintering (SLS), and FDM (also referred to as fused filament fabrication, FFF), as illustrated in Figure 2 (Khorsandi et al., 2021). SLA and SLS techniques are respectively categorized as vat photopolymerization and PBF, utilizing liquid resin and powder as their raw materials. The SLA system can achieve intricate internal features and enable the growth factor, protein, and cell patterning by 1) immersing the platform in the photopolymer liquid, 2) exposing it to the focused light based on the desired design, 3) solidifying the polymer at the focal point while keeping the non-exposed polymer in liquid form, and 4) fabricating objects layer-by-layer as the platform moves downward. However, this method is only suitable for photopolymers (Bose et al., 2013). A different technique of 3D printing, known as SLS, involves using a powerful energy source like a laser to melt PEEK and to build objects with melting substances via layer-by-layer addition of powder and sintering each layer in the 3D printer based on the CAD file (Lau et al., 2020). FDM, a printing method that involves material extrusion, extrudes strands of melted polymer through a heated nozzle onto the workbench in a sequential manner (Chen et al., 2022).

To facilitate the understanding of PEEK concerning bone reconstruction, this part outlines the advantages and disadvantages of representative 3D printing techniques for PEEK, with a focus on the consolidation of extruded layers, referred to as FDM, and the fusion of polymer powder, known as SLS. The production of PEEK constructs using FDM has been explained, offering numerous benefits, including the ability to perform personalized and swift production in small quantities, reduced material wastage, improved cost-efficiency, and simplified operation (Feng et al., 2020; Luo et al., 2023). Nevertheless, the physical characteristics of FDM printed polymers frequently fail to satisfy the demands of applications because of the weak bonding between layers. The extensive utilization of FDM printed PEEK is also restricted due to its high melting (334°C) temperature and glass transition (143°C) temperature, high viscosity, semicrystalline nature, and significant shrinkage post-cooling, in contrast to conventional low-crystalline polymers employed in fused modeling-based 3D printing (Chen et al., 2019; He et al., 2021; Manzoor et al., 2021).

Although SLS allows for the rapid creation of intricate constructs and offers excellent flexibility in material options, its ability to produce detailed features relies on the laser beam diameter, while concerns arise regarding the sterilization and safety of powder management during SLS printing in a hospital setting (Basgul et al., 2021). In contrast, the FDM medical system can accomplish printing in a sterile environment. The cost of purchasing an SLS system and its high energy requirements should also be considered. SLS printing is limited by the unfavorable mechanical properties of printed PEEK constructs due to the non-isothermal crystallization of semicrystalline polymer powder during the entire processing of PBF (Chen et al., 2020b).

The utilization of 3D printing has also been expanded to produce composites like PEEK/carbon fiber and PEEK/HAP scaffolds (Han et al., 2019; Manzoor et al., 2021). However, the printing of these composites, for instance, the FDM printed PEEK/HAP composite, is more difficult than pure PEEK because of the presence of HAP fillers that alter the viscoelastic and thermo-mechanical properties of the matrix. Despite its benefits, 3D printing is impractical for mass production due to its expansive cost and limited efficiency. Furthermore, the limited resolution achieved in 3D printing to date poses a challenge to accurately fabricating constructs with small-scale or arbitrary structures (He et al., 2021). And the tensile strength of 3D printed constructs is generally lower compared to those produced through injection molding, primarily attributed to the weak bonding between layers and the existing empty spaces (Manzoor et al., 2021). Research results indicated that the tensile strength of FDM printed PEEK ranged from 46% to 97% of injection molded samples (Basgul et al., 2021). However, a recent study discovered that the tensile strength of FDM printed PEEK was 95.21 ± 1.86 MPa with an elastic modulus of 3.79 ± 0.27 GPa, similar to the injection molded pure PEEK (100 MPa and 4 GPa) (Han et al., 2019). For another printing technique, compared to dog bone samples of SLS printed PEEK with nano additives (Al_2_O_3_, TiO_2_, ZrO_2,_ or HAP), SLS printed pure PEEK achieved the highest strength (33 MPa), which was 34% of the strength of samples produced by injection molding (Shishkovsky et al., 2018).

Evaluating the mechanical features, accuracy, and precision of printed PEEK under different printing conditions using different printing techniques is critical in research, as appropriate adjustment of printing parameters such as nozzle temperature and print orientation has been reported to yield FDM printed PEEK constructs with mechanical properties (e.g., tensile strength, flexural strength, and impact strength) that reach around 80% of those of injection molded parts (Ding et al., 2019). Different properties of 3D printed PEEK can be achieved by adjusting factors like printing temperature, power energy, printing speed, and layer thickness. The nozzle temperature is considered the most significant factor influencing the tensile strength of FDM printed PEEK in comparison to printing speed and layer thickness (Timoumi et al., 2022). A study on FDM printed PEEK composites with 5 wt% carbon fiber indicated that the tensile and flexural strength of PEEK/carbon fiber constructs showed improvement as the nozzle and platform temperatures increased, possibly attributed to the enhanced flow and formability of the material at elevated nozzle temperature (Wang et al., 2021). Furthermore, the elevated platform temperature may produce additional energy for enhanced infiltration and diffusion among filaments and interlayers. Basgual et al. (2021) reported that the nozzle temperature for FDM printing varied from 340°C to 485°C, while the printing bed temperature fell within the range of 100°C–250°C. And the environment/chamber temperatures ranged from 20°C to 200 °C. It is essential to maintain an appropriate speed to rapidly manufacture 3D printed constructs without compromising their biomechanical strength, where a printing speed ranging from 5 to 50 mm/s was typically applied in FDM studies. The nozzle diameter in FDM and the spot diameter in SLS are additional crucial elements in determining the layer height and influencing the overall mechanical results. As for FDM, the layer height is anticipated to be around 25%–50% of the nozzle diameter, whereas the spot diameter for SLS frequently ranged from 0.4 to 3.5 mm, with 0.8 mm being the most prevalent diameter. In SLS studies, the laser power ranged between 1.9 and 28 W, while the printing speed spanned from 2.1 to 5080 mm/s.

## 4 Improved 3D printed PEEK for bone reconstruction

In addition to its use in fracture fixation, maxillofacial bone reconstruction, occlusal splints, and implant surgical guides, PEEK is also commonly processed into three-dimensional porous scaffolds to treat massive bone defects (Zhang et al., 2019; Li et al., 2022a). And 3D printed PEEK constructs can be customized for use in complicated cranioplasty, orthopedic surgery, dentistry, and other fields. However, despite their wide use in biomedical fields, these 3D printed PEEK constructs suffer from disadvantages such as insufficient mechanical strength, biological inertness, and poor antibacterial ability. Therefore, various modifications can be adapted to improve their performance for bone reconstruction (AlOtaibi et al., 2020).

### 4.1 Mechanically improved 3D printed PEEK designs

The stiffness of PEEK may be insufficient to withstand loading (Wang et al., 2022a), and mandibular reconstruction using CAD/CAM PEEK plates, without the reinforcement of carbon fiber, has been reported to fail in ensuring displacement and mechanical stability as effectively as miniature titanium plates (Steffen et al., 2020). Therefore, it is critical to use various methods, including the incorporation of additional substances such as fibers, ceramics, and nanoparticles, and the application of post-treatment, to improve the mechanical performance of 3D printed PEEK.

**FIGURE 3:**
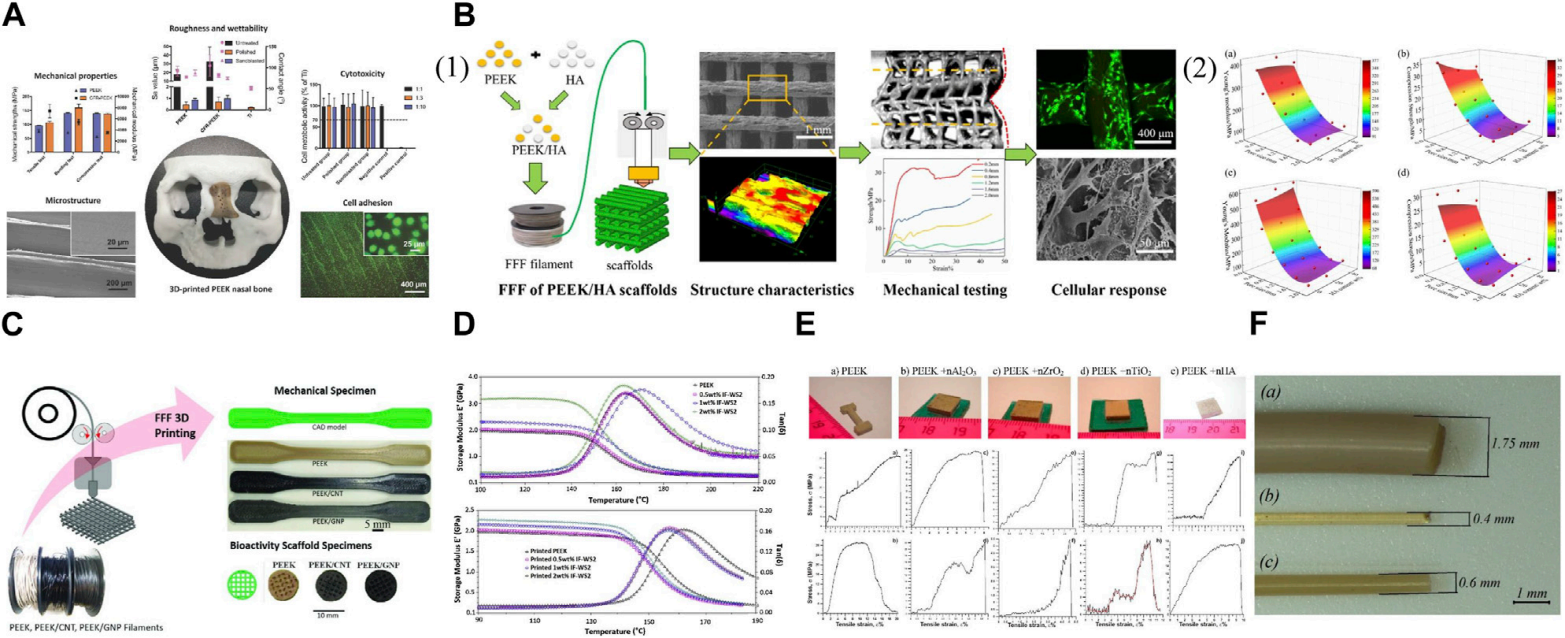
Mechanically improved 3D printed PEEK designs. **(A)** FDM printed PEEK reinforced with carbon fiber. Reproduced with permission (Han et al., 2019). Copyright 2019, MDPI. **(B) (1)** FDM printed PEEK/hydroxyapatite porous scaffolds. **(2)** The modulus and strength varied with pore size and HA content: **(a)** modulus and **(b)** strength compressed along Z printing direction, **(c)** modulus and **(d)** strength compressed along X printing direction. Reproduced with permission (Zheng et al., 2021). Copyright 2021, Elsevier. **(C)** Nanoengineered PEEK filaments are used to 3D print (via FFF) mechanical dogbone and bioactivity scaffold specimens. Reproduced with permission (Alam et al., 2020). Copyright 2020, The Authors, published by WILEY-VCH Verlag GmbH and Co. KGaA, Weinheim. **(D)** Storage modulus (E′) and Loss tangent (tanδ) vs. temperature for injection molded PEEK samples **(above)** and printed PEEK samples **(below)**. Reproduced with permission (Golbang et al., 2020). Copyright 2019, Elsevier. **(E)** Appearance of samples after the SLS process **(above)**. The stress-strain diagrams of the 3D printed samples from PEEK with nano additives **(below)**: **(a,b)** pure PEEK; **(c,d)** PEEK + nAl_2_O_3_; **(e,f)** PEEK + nZrO_2_; **(g,h)** PEEK + nTiO_2_; **(i,j)** PEEK + nHA. The upper row **(a,c,e,g,i)**, the measurements were at room temperature, the lower row **(b,d,f,h,j)** at heating to −50°C. Reproduced with permission (Shishkovsky et al., 2018). Copyright 2018, Elsevier. **(F) (a)** As-delivered filament from coil; **(b)** filament extruded via 0.4 mm nozzle before heat treatment; and **(c)** filament extruded via 0.6 mm nozzle after heat treatment. Reproduced with permission (Vindokurov et al., 2022). Copyright 2022, The Authors, Licensee MDPI, Basel, Switzerland.

Carbon fibers can be mixed with 3D printed PEEK for enhanced mechanical properties. Samples of FDM printed PEEK reinforced with carbon fiber (CFR-PEEK) exhibited significantly greater overall mechanical strength compared to pure PEEK samples, while both surface-modified and unmodified PEEK and CFR-PEEK exhibited excellent cytocompatibility *in vitro* (Figure 3A) (Han et al., 2019). Therefore, this study demonstrated that the FDM fabricated CFR-PEEK composite with sufficient mechanical properties may be a promising biomaterial for biomedical bone grafting and tissue engineering. Because the FDM process for fiber-reinforced PEEK composites requires high thermal conditions (380°C–440 °C), a significant increase in melt viscosity, leading to failure in 3D printing may occur if PEEK/carbon fiber composites have a fiber content exceeding 20 wt%. Short and continuous fiber-reinforced composites are extensively suggested in FDM to enhance the mechanical and thermal characteristics of polymer-based materials (Wang et al., 2021).

Through the manipulation of pore dimensions and HAP concentration, the elastic modulus of PEEK/HAP composite scaffolds fabricated using FDM printing could be extensively adjusted within the span of 50.6–624.7 MPa, resembling the variation observed in natural cancellous bone (Figure 3B) (Zheng et al., 2021). More accurate predictions and control over the mechanical modulus and strength of final scaffolds could be achieved based on the established correlation among geometric parameters, HAP content, printing direction, and mechanical properties. In addition, the micro-structured surface of these composite scaffolds was reported to facilitate cell adhesion and mineralization *in vitro*. However, contrary to the rise in compressive strength and modulus, research indicated that the tensile and flexural strength decline as the HAP content increases (Li et al., 2021a; Zheng et al., 2021). Therefore, the load-bearing capability of 3D printed PEEK constructs reinforced with bioactive ceramics may be compromised compared to other composites. To tackle this problem, carbon nanostructures, including carbon nanotubes and graphene nanoplatelets, have been investigated to offer an additional option for reinforcing AM PEEK in the creation of bioactivated surfaces as bone scaffolds, with further potential arising from the electrically conductive nanoengineered PEEK for the development of intelligent and versatile structures (Figure 3C) (Alam et al., 2020). PEEK/HAP composites fabricated using FDM pose challenges compared to pure PEEK because HAP may modify the viscoelastic and thermomechanical properties of the matrix (Manzoor et al., 2021).

To reduce the deformation of the printed constructs, techniques like incorporating enhanced technology to include additives for minimizing crystallization and diluting the crystalline substance with fillers have been employed, especially considering that 3D printing favors the utilization of glassy polymers over semicrystalline ones as the latter experience a step change in viscosity when they crystallize, leading to a halt in polymer mobility and deformation of the printed constructs with the generated stresses (Luo et al., 2023). The incorporation of inorganic fullerene tungsten sulfide nanoparticles (IF-WS_2_) into PEEK through melt compounding improved the polymer’s flowability and decreased its melt viscosity by 25% while maintaining mechanical and thermal characteristics (Figure 3D) (Golbang et al., 2020). Subsequently, the FDM printing quality of the PEEK nanocomposite filaments and the mechanical properties of printed PEEK were improved. These findings indicated that the incorporation of IF-WS_2_ nanoparticles into PEEK may be useful for creating high-performance material for the FDM process, especially as PEEK’s high viscosity can lead to filament buckling in FDM. However, SLS printed PEEK with various nano additives (Al_2_O_3_, TiO_2_, ZrO_2_, or HAP) showed decreased strength and deformation properties of final parts compared to pure PEEK (Figure 3E) (Shishkovsky et al., 2018). Therefore, it is advisable to blend nano-additives with PEEK cautiously, as the incorporation of fillers in the scaffolds to increase mechanical strength is reasonable when the fillers do not exceed the threshold, which depends on the ratio of the composite to the incorporated substance. Filler size can also affect mechanical properties.

The post-treatment of filaments can influence the mechanical characteristics of PEEK. A study discovered that subjecting the filament to a heated treatment (at a temperature of 220°C) after printing enhanced the interlayer strength (Figure 3F) (Vindokurov et al., 2022). As a result, the elastic modulus of FDM printed PEEK samples improved by 20%, while the tensile strength increased by 45%–65% and the fracture resistance by 33%–45%.

### 4.2 Biologically improved 3D printed PEEK designs

**FIGURE 4:**
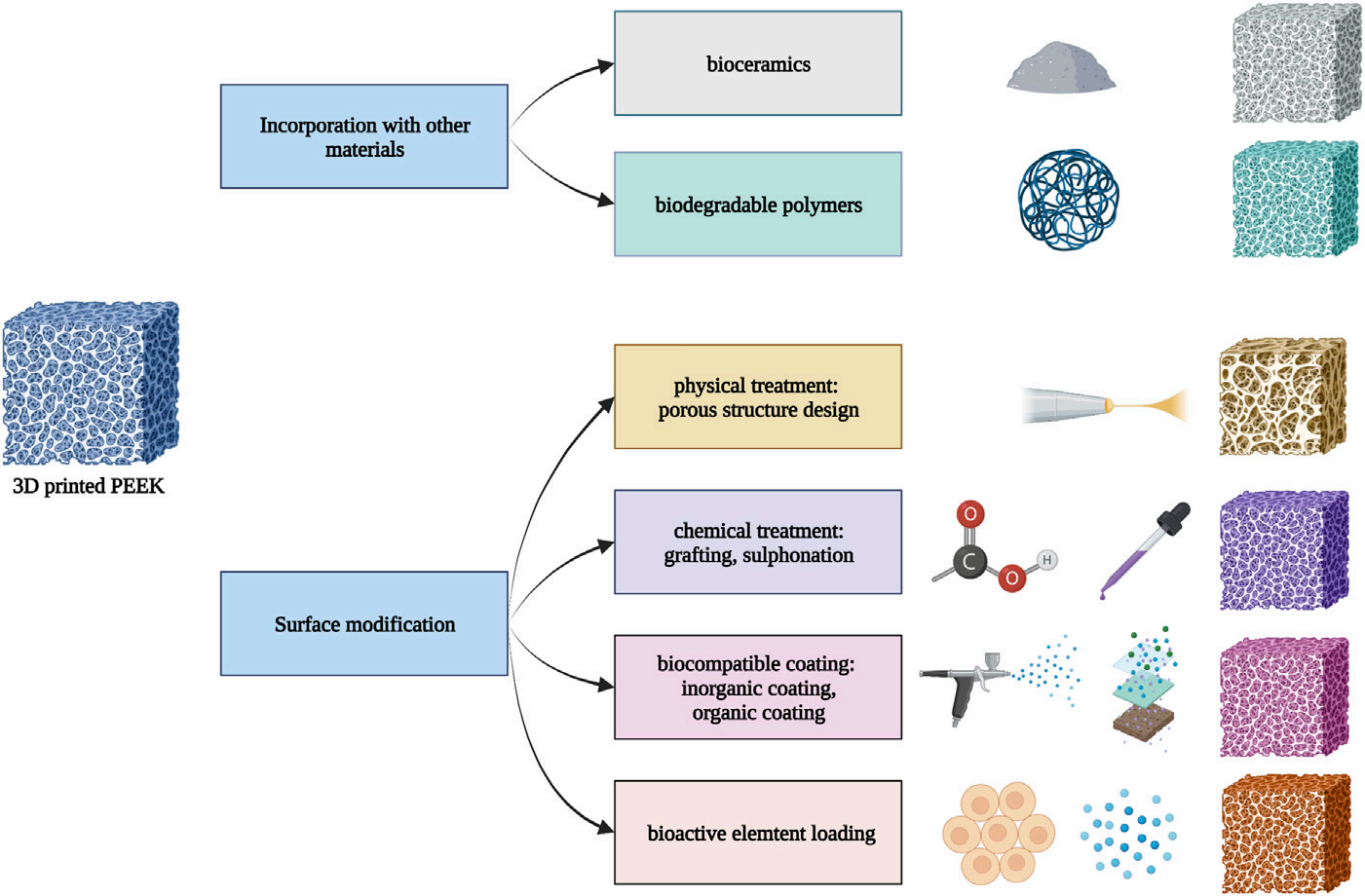
Modified strategies to improve the biological performance of 3D printed PEEK.

This section outlines two primary approaches to improving the interactions between bone tissue and 3D printed PEEK: incorporation of other materials and surface modification. First, the incorporation process involves combining PEEK with ceramics, carbon fiber, glass fiber, or polymers to create PEEK composites using 3D printing technology. Second, some of the surface treatment methods for PEEK, including physical treatment (plasma immersion, physical vapor deposition, porous or roughened structure design, etc.), chemical treatment (sulfonation, grafting, etc.), biocompatible coating (graphene oxide coating, HAP coating, polydopamine (PDA) coating, etc.), and loading of bioactive elements, can also be applied to modify 3D printed PEEK (Figure 4).

#### 4.2.1 Incorporation with other materials

**FIGURE 5:**
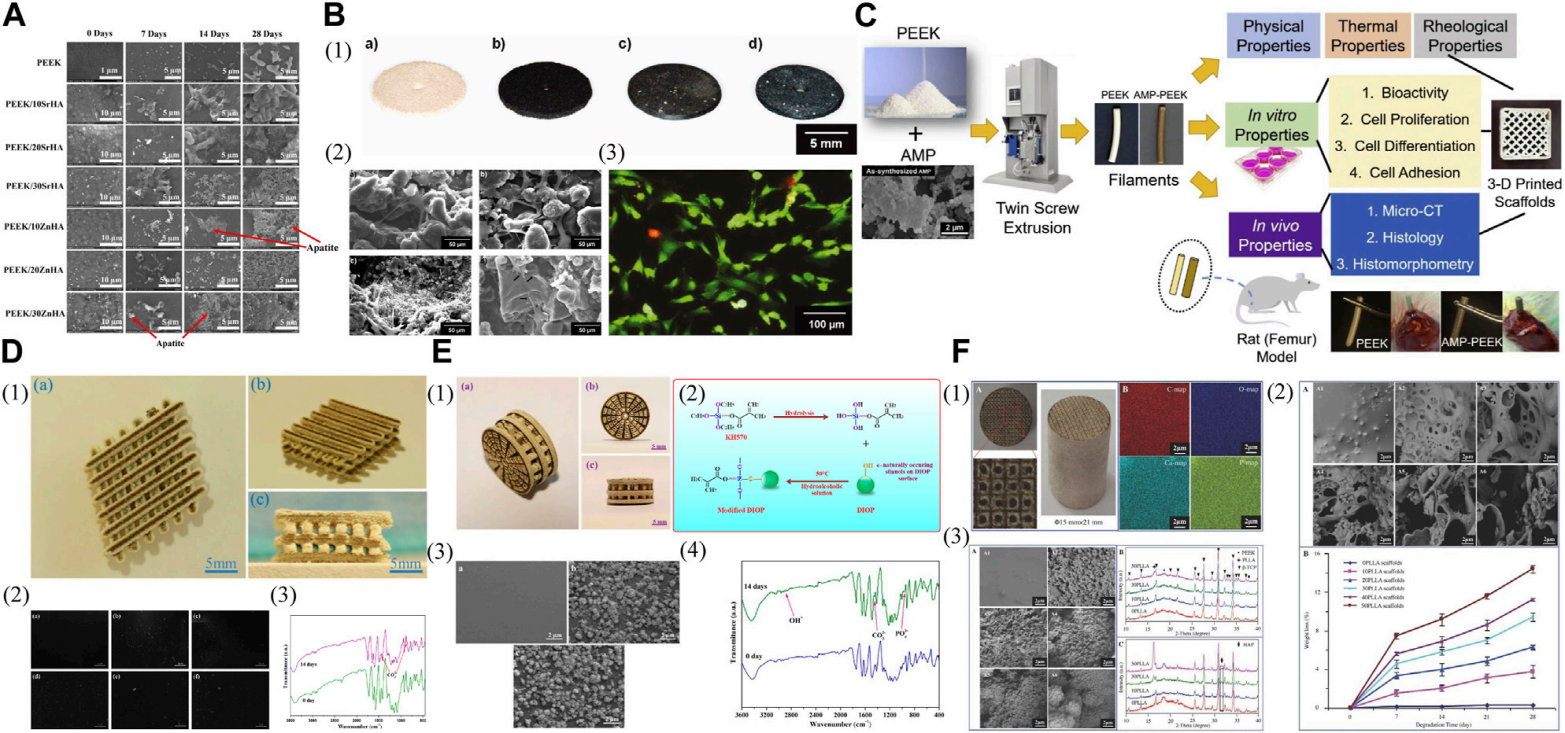
Incorporation with other materials for biologically improved 3D printed PEEK constructs. **(A)** SEM images showing the apatite layer formation on the samples of PEEK and its nanocomposites with Strontium doped hydroxyapatite (SrHA) and Zinc doped hydroxyapatite (ZnHA) after immersion in SBF for 0, 7, 14, and 28 days. Reproduced with permission (Manzoor et al., 2022). Copyright 2022, The Authors, Licensee MDPI, Basel, Switzerland. **(B)** **(1)** Laser sintered PEEK cell test specimens. **(a)** PEEK pure, **(b)** PEEK/1 wt% carbon, **(c)** PEEK/1 wt% carbon/10 wt% β-TCP, **(d)** PEEK/1 wt% carbon/10 wt% Bioglass. **(2)** SEM pictures of sample surfaces with human osteoblasts. **(a)** PEEK pure, **(b)** PEEK/1 wt% carbon, **(c)** PEEK/1 wt% carbon/10 wt% β-TCP, **(d)** PEEK/1 wt% carbon/10 wt% Bioglass. **(3)** FDA/PI viability staining of osteoblasts growing on a PEEK pure sample for 24 h. Reproduced with permission (von Wilmowsky et al., 2008). Copyright 2008, Wiley Periodicals, Inc. **(C)** Schematic illustration of the fabrication steps as well as physical, thermal, rheological, and biological characterizations of AMP-PEEK composite filaments for 3D printing applications. Reproduced with permission (Sikder et al., 2020). Copyright 2020, The Academy of Dental Materials. Published by Elsevier Inc. All rights reserved. **(D) (1) (a)** Top view; **(b)** isometric view; and **(c)** lateral view of the polyetheretherketone/polyglycolicacid-hydroxyapatite (PEEK/PGA-HAP) composite scaffold. **(2)** Morphologies of the scaffolds with **(a)** 0 wt%; **(b)** 5 wt%; **(c)** 7.5 wt%; **(d)** 10 wt%; **(e)** 12.5 wt%; **(f)** 15 wt% HAP after immersion in simulated body fluid (SBF) for 14 days. **(3)** Fourier transform infrared spectrums of the scaffolds with 10 wt% HAP before and after immersing in SBF. Reproduced with permission (Shuai et al., 2016b). Copyright 2016, MDPI. **(E) (1) (a)** Lateral view; **(b)** front view; and **(c)** isometric view of the PEEK/PGA-KDIOP composite scaffold. **(2)** Schematic of silane reaction to produce the surface modified DIOP particles. **(3)** SEM micrographs of SEM micrographs of **(a)** PEEK/PGA; **(b)** PEEK/PGA-10% DIOP; and **(c)** PEEK/PGA-10% KDIOP scaffolds after immersion in simulated body fluid (SBF) for 14 days. **(4)** Fourier transform infrared spectrums of the scaffolds with 10% KDIOP after immersion in SBF. Reproduced with permission (Shuai et al., 2017). Copyright 2017, MDPI. **(F)**. **(1)** The characteristics of scaffolds. **A)** Optical graphs. **B)** EDS mapping images. **(2)** The degradation behaviors of the scaffolds after PBS immersion. **A)** SEM micrographs of the scaffolds with 0-50 wt% of PLLA content (A1-A6) after PBS immersion for 28 d. **B)** Weight loss of the scaffolds with 0-50 wt% of PLLA content as a function of degradation time. **(3)** The bioactivity of the scaffolds with 0-50 wt% of PLLA content before and after SBF immersion for 28 d. **A)** SEM micrographs of the scaffolds (A1-A6) after SBF immersion. **B)** XRD patterns before SBF immersion. **C)** XRD patterns after SBF immersion. Reproduced with permission (Feng et al., 2018). Copyright 2018, The Authors. published by WILEY-VCH Verlag GmbH and Co. KGaA, Weinheim.

HAP is a calcium phosphate found naturally in human bones and makes up most of the inorganic elements. The FDM printed PEEK nanocomposites that contained 20 wt% of strontium (Sr) and zinc (Zn) doped-HAP nanoparticles and then were deposited with polyethylene glycol-1–3,4-dihydroxyphenylamine (PEG-DOPA) may be the optimal choice for the 3D printed craniomaxillofacial implants due to their exceptional mechanical properties, desirable apatite formation, and enhanced hydrophilicity (Figure 5A) (Manzoor et al., 2022).

Other calcium phosphate-rich fillers, such as β-tricalcium phosphate (β-TCP) and bioactive glass with a composition resembling HAP, have also been utilized to modify 3D printed PEEK. The SLS produced PEEK/carbon black composites with bioactive glass 45S5 or β-TCP as bioactive enhancement particles seem to be attractive candidates as substitutes for bone reconstructive surgery (Figure 5B) (von Wilmowsky et al., 2008). Compared to the control group (pure PEEK samples), the samples containing bioactive glass demonstrated the highest osteoblast proliferation and viability *in vitro* and in contrast, the samples containing β-TCP exhibited the lowest. This study suggested that compounding bioinert PEEK powder with osteoconductive and bioactive materials may further benefit bone tissue formation *in vivo*.

Other phosphates have also been deployed as substitutes for modifying 3D printed PEEK to enhance bone-implant integration *in vivo*. Amorphous calcium phosphate (ACP) tends to undergo crystallization, whereas amorphous magnesium phosphate (AMP) through the inclusion of Mg^2+^ ions in ACP can prevent the crystallization and exhibit thermal stability. AMP-PEEK composite filaments for 3D printing, created through melt-blending AMP particles with PEEK, exhibited increased bioactivity and favorable pre-osteoblast cell-related results *in vitro* as compared to bare PEEK, the bone integration ability of which was further enhanced as evidenced by *in vivo* results (Figure 5C) (Sikder et al., 2020). These composite filaments also demonstrated a high zero-shear viscosity and low infinite-shear viscosity, holding potential as bioactive feedstock for 3D printing.

Nevertheless, the incorporation of bioactive ceramics can lead to fatigue damage in the composites and interfacial failure due to inadequate adhesion, resulting in the initiation and propagation of matrix cracks originating from the bonding site between the filler and matrix (Gao et al., 2022).

For optimal bone healing, the biodegradation rate should align with that of the host tissue (Hu et al., 2022; Hu et al., 2023c). Therefore, biodegradable polymers like poly (glycolic acid) (PGA) can be deployed to modify printed PEEK. SLS printed porous PEEK/PGA composite scaffolds showed an improved and controllable degradation rate by adjusting the PGA content while maintaining biocompatibility and suitable mechanical properties (Shuai et al., 2016a). This study indicated that the breakdown of PGA could enhance cellular adhesion and growth, and the ability to control the degradation rate enabled PEEK to offer adequate strength in specific regions. To enhance their performance further, Shuai et al. (2016a) integrated HAP into a composite of PEEK/PGA matrix and then developed SLS printed composite scaffolds that showcased the promise for tissue regeneration (Figure 5D). These PEEK/PGA composite scaffolds containing 10 wt% of HAP yielded the best results, enhancing cell attachment and proliferation more effectively compared to PEEK/PGA scaffolds without HAP. The SLS printed porous PEEK/PGA/KDIOP composite scaffolds, which contained 3-glycidoxypropyltrimethoxysilane (KH570, a silane coupling agent)-modified DIOP (a calcium magnesium silicate bioceramic) (KDIOP), demonstrated the ability to generate apatite *in vitro* and cell culture tests showed favorable cytocompatibility of them when compared to scaffolds that did not include KDIOP (Figure 5E) (Shuai et al., 2017). Nevertheless, the compatibility performance of PEEK/PGA-10% DIOP samples was comparable, and there was no indication of a distinction between the addition of DIOP or KDIOP. By combining biodegradable poly (l-lactic acid) (PLLA) with a composite of PEEK and β-TCP, multi-material composite scaffolds were created using SLS (Figure 5F) (Feng et al., 2018). These scaffolds exhibited excellent bioactivity, biodegradability, and cytocompatibility, and after being implanted *in vivo* for 8 weeks, the defected region was fully integrated with the surrounding host bone.

#### 4.2.2 Surface modification

**FIGURE 6:**
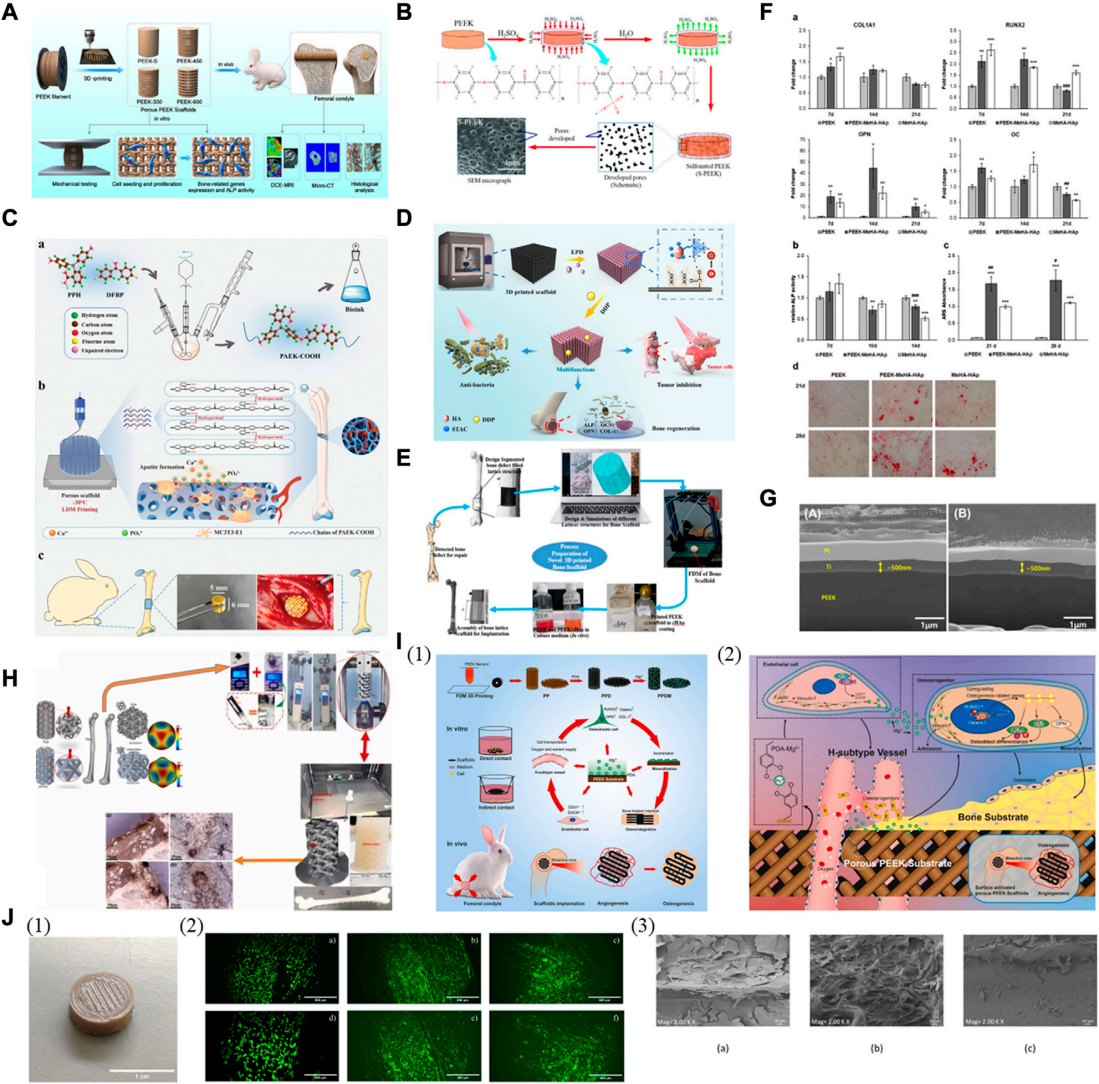
Surface modification for biologically improved 3D printed PEEK constructs. **(A)** Schematic illustration of the fabrication of porous PEEK scaffolds and their bioapplication. Reproduced with permission (Feng et al., 2020). Copyright 2020, American Chemical Society. **(B)** Schematic diagram of sulfonation process of PEEK and its nanocomposite samples processed by FFF 3D printing. Reproduced with permission (Alam et al., 2020). Copyright 2020, The Authors, published by WILEY-VCH Verlag GmbH and Co. KGaA, Weinheim. **(C)** Schematic elucidating the fabrication of hierarchically porous PAEK-COOH scaffold and the process promoting bone formation. **(a)** Synthetic process of PAEK-COOH and preparation of bioink. **(b)** The hierarchically porous scaffold of LDM-printed PAEK-COOH favoring cellular adhesion and HA mineralization. **(c)** Implanted scaffold inducing bone formation *in vivo*. Reproduced with permission (Gao et al., 2022). Copyright 2022, Wiley-VCH GmbH. **(D)** Schematic showing scaffold preparation and its associated multi-functions. Reproduced with permission (Zhu et al., 2021). Copyright 2021, American Chemical Society. **(E)** Process of a bone implant in fused deposition modeling via *in vitro*. Reproduced with permission (Oladapo et al., 2020). Copyright 2020, Elsevier B. V. All rights reserved. **(F)** Osteogenic differentiation on PEEK-MeHA-HAp, MeHA-HAp, and PEEK. **(a)** Expression profile of osteogenesis-related genes: COL1A1, RUNX2, OPN, and AC. **(b)** Alkaline phosphatase (ALP) activity. **(c)** Alizarin Red S (ARS) quantification and **(d)** staining. Reproduced with permission (Ferroni et al., 2022). Copyright 2022, The Authors. Licensee MDPI, Basel, Switzerland. **(G)** Cross-sectional morphologies of Ti coatings on **(A)** machined PEEK and **(B)** 3D-printed PEEK. Cross-sectional morphologies of Ti coatings on **(A)** machined PEEK and **(B)** 3D-printed PEEK. Reproduced with permission (Jung et al., 2019). Copyright 2019, IOP Publishing Ltd. **(H)** The fabrication and *in-vitro* biocompatibility of FDM printed PEEK scaffolds coated with reduced graphene oxide (rGO). Reproduced with permission (Oladapo et al., 2022). Copyright 2022, The Authors, published by Elsevier B. V. **(I) (1)** The fabrication and *in-vitro* and *in-vivo* characterization of magnesium surface-activated 3D-printed porous PEEK scaffolds coated with polydopamine. **(2)** The mechanism by which magnesium surface-activated 3D-printed porous PEEK scaffolds promote angiogenesis and osteogenesis. Reproduced with permission (Wei et al., 2023). Copyright 2022, The Authors. publishing services by Elsevier B. V. on behalf of KeAi Communications Co. Ltd. **(J) (1)** Patterned surface of 3D-printed PEEK disk. **(2)** Live and dead staining of human osteoblasts cultured for 48 h on non-functionalized **(a,d)** and functionalized PEEK (through amino-oxy **(b,e)**; through azido groups **(c,f)**. Very few dead cells (in red) are seen in **(f)** in contrast to a large number of viable cells (in green) in all the images **(a–f)**. **(3)** Scanning electron microscopy images of human osteoblast cells 48 h after seeding. The samples shown are the unfunctionalized PEEK sample **(a)** and PEEK functionalized with GBMP1α through amino-oxy **(b)** and azido groups **(c)**. (1) Patterned surface of 3D-printed PEEK disk. (2) Live and dead staining of human osteoblasts cultured for 48 h on non-functionalized **(A,D)** and functionalized PEEK (through amino-oxy **(B,E)**; through azido groups **(C,F)**). Very few dead cells (in red) are seen in **(F)** in contrast to a large number of viable cells (in green) in all the images **(A–F)**. (3) Scanning electron microscopy images of human osteoblast cells 48 h after seeding. The samples shown are the unfunctionalized PEEK sample **(A)** and PEEK functionalized with GBMP1α through amino-oxy **(B)** and azido groups **(C)**. Reproduced with permission (Cassari et al., 2023). Copyright 2023, The Authors. Licensee MDPI, Basel, Switzerland.

The porous PEEK surface with interconnected porous structure through physical treatment can promote interface interlocking and osseointegration, as supported by the positive *in vitro* and *in vivo* results of the FDM printed porous PEEK scaffolds designed with a suitable pore size (Figure 6A) (Feng et al., 2020). Apart from the established sulfonation process detailed in the following part, 3D printing is also an effective way to create a porous structure with highly interconnected pores within PEEK constructs.

Introducing charged sulfonate (-SO_3_H) groups into the polymer using corrosive acids can increase hydrophilicity and create a porous structure. However, the presence of residual components and an abundance of sulfur functional groups can potentially damage host cells and tissues, although sulfonated PEEK samples processed by hydrothermal treatment to remove excess sulfur have been reported to exhibit enhanced bone integration and antibacterial effects both *in vitro* and *in vivo* (Ouyang et al., 2016). Further investigation is expected for 3D printed sulfonated PEEK for bone regeneration, although research has demonstrated the beneficial chemical and morphological effects of modifying the surface of PEEK constructs with 98% sulfuric acid due to the formed sulfonate groups within the highly porous and permeable layer, as well as the increased contact surface area (Chaijareenont et al., 2018), and that FDM printed porous sulfonated PEEK scaffolds have the potential to enhance functional cartilage repair (Yuan et al., 2022). Carbon nanostructures incorporated into PEEK to form composite scaffolds via FDM showed favorable effects on structural performance, along with the synergistic results of carbon nanostructure incorporation and sulfonation treatment for enhanced bioactivity (Figure 6B) (Alam et al., 2020).

Grafting functional groups onto the surface is also helpful, yet sometimes too laborious and problematic to regulate. A study of low-temperature 3D printing for the fabrication of hierarchically controllable porous scaffolds using the synthesized bioink of amorphous poly (aryl ether ketone) (PAEK) and carboxyl groups (PAEK-COOH) indicated that these scaffolds exhibited mechanical strength comparable to that of the trabecular bone (Figure 6C) (Gao et al., 2022). *In vitro*, the nanoporous surface promoted cellular adhesion, while the carboxyl groups facilitated HAP mineralization through electrostatic interactions. Compared to PEEK, these PAEK-COOH scaffolds showed better *in vivo* integration with bone tissue without additional active ingredients.

Various types of inorganic bioactive coatings for enhanced osseointegration of 3D printed PEEK have been utilized. To begin with, a PEEK/graphene nanocomposite scaffold was created using FDM printing and then deposited with HAP containing antibiotics and/or anti-cancer drugs, aiming to realize multimodal treatment therapy for bone diseases like osteosarcoma and osteomyelitis (Figure 6D) (Zhu et al., 2021). The surface modification with the bioactive HAP coating resulted in a remarkable increase in the proliferation of stem cells *in vitro* and facilitated the growth of new bone *in vivo*. The existence of antibiotics and anti-cancer medications allowed for the elimination of bacteria resistant to drugs and the removal of cancer cells and the treatment performance can be improved even more with laser-induced heating when needed. The microporous composite scaffold formed by coating FDM printed PEEK with calcium hydroxyapatite (cHAP) Ca_10_(OH) (PO_4_)_3_ induced apatite formation after immersion in the simulated body fluid with superior osseointegration and biological activity than bare PEEK *in vitro* (Figure 6E) (Oladapo et al., 2020). It is recognized that the permeable surface can enhance the ingrowth of bone tissue and the stability of implants *in vivo*, especially advantageous for the long-term durability of bone implants, as evidenced by the study that the porous PEEK scaffolds created through FDM printing promoted osteointegration at the femoral condyle of rabbits more effectively than the solid group, the process of which was further enhanced by applying a HAP coating to the scaffolds (Wu et al., 2023). The combination of a 3D printed interconnected porous PEEK scaffold and a methacrylated hyaluronic acid (MeHA)-HAP hydrogel coating, forming a hybrid bone substitute, enhanced the attachment and growth of human mesenchymal stem cells (MSCs) and facilitated osteogenic differentiation and ECM mineralization *in vitro*, showing great promise for future clinical use (Figure 6F) (Ferroni et al., 2022).

Titanium oxide (TiO_2_) is another frequently employed ceramic coating with bioactive nature. PEEK and its derivative, poly (ether ketone ketone) (PEKK), have been utilized as implant materials. FDM printed porous PEKK bone analogs with a designed optimized structure and a bioactive titanium oxide coating may enhance bone regeneration for mandibular reconstruction, as *in vivo* analyses demonstrated improved bone ingrowth within these analogs (Cheng et al., 2023a).

The application of a biocompatible metallic coating can improve the bioactivity of 3D printed PEEK. A layer of titanium was deposited on FDM printed PEEK implants to improve the interfacial biocompatibility, and it was found to significantly enhance their ability of bone regeneration *in vitro* and *in vivo* (Figure 6G) (Jung et al., 2019).

The inorganic coating made from graphene nanomaterials and their derivatives is utilized for 3D printed PEEK. The application of a reduced graphene oxide (rGO) coating on FDM printed PEEK (PEEK-rGO) demonstrated the great promise of PEEK-rGO scaffolds as implants with *in-vitro* biocompatibility for biomimetic heterogeneous bone repair (Figure 6H) (Oladapo et al., 2022).

In terms of organic coating, PDA, a polymer created through oxidating dopamine, is considered a desirable substance for forming coatings rich in catechols, amines, and quinones which can facilitate robust bonding with molecules (e.g., peptides), nano HAP, and metal ions. Furthermore, PDA-coated PEEK can covalently bond collagen and insulin, leading to a significant improvement in bioactivity (Kwon et al., 2018). Regarding 3D printed PEEK, it is possible to modify FDM printed porous PEEK scaffolds by applying a PDA coating chelated with magnesium ions (Mg^2+^) (Figure 6I) (Wei et al., 2023). The stimulated surface improved cellular proliferation and adhesion *in vitro*, thereby supporting osteoblast differentiation and mineralization, and Mg^2+^ ions stimulated angiogenesis, facilitating the formation of osteogenic type H vessels. The *in vivo* findings demonstrated that the personalized porous structure promoted the ingrowth of vessels and bone within these PEEK scaffolds, where the PDA coating improved the interfacial osseointegration and Mg^2+^ expedited the initial bone ingrowth by stimulating the early formation of blood vessels during the coating degradation.

Combining 3D printed PEEK with cell therapy shows potential for treating bone defects, as functionalizing bioactive polymers like PEKK with adipose-derived stem cells (ADSCs) enhanced bone tissue integration and bone formation of 3D printed PEKK/ADSCs implants placed in critical-sized rabbit mandibular defects (Roskies et al., 2017; Li et al., 2021b). Researchers modified the internal structure to form a trabecular network and impregnated SLS printed porous PEEK with MSCs (Roskies et al., 2016). The obtained PEEK scaffolds preserved the viability of adipose- and bone marrow-derived MSCs while encouraging better osteogenic differentiation of adipose-derived MSCs than bone marrow-derived MSCs. Furthermore, through the covalent anchoring of a peptide that mimics bone morphogenetic protein-2 (BMP-2) to the FDM printed PEEK, these functionalized samples exhibited more excellent cell coverage without cytotoxicity than the control and led to an increase in cell proliferation rate and a higher quantity of calcium deposits (Figure 6J) (Cassari et al., 2023).

### 4.3 Antibacterial 3D printed PEEK designs

Surgical site infection is one of the most common complications from implanted devices and one of the most critical factors in early bone-implant failure. Until now, extensive research has been conducted to impart antibacterial capacity to 3D printed PEEK to prevent the initial attachment of bacteria and the development of biofilms.

**FIGURE 7:**
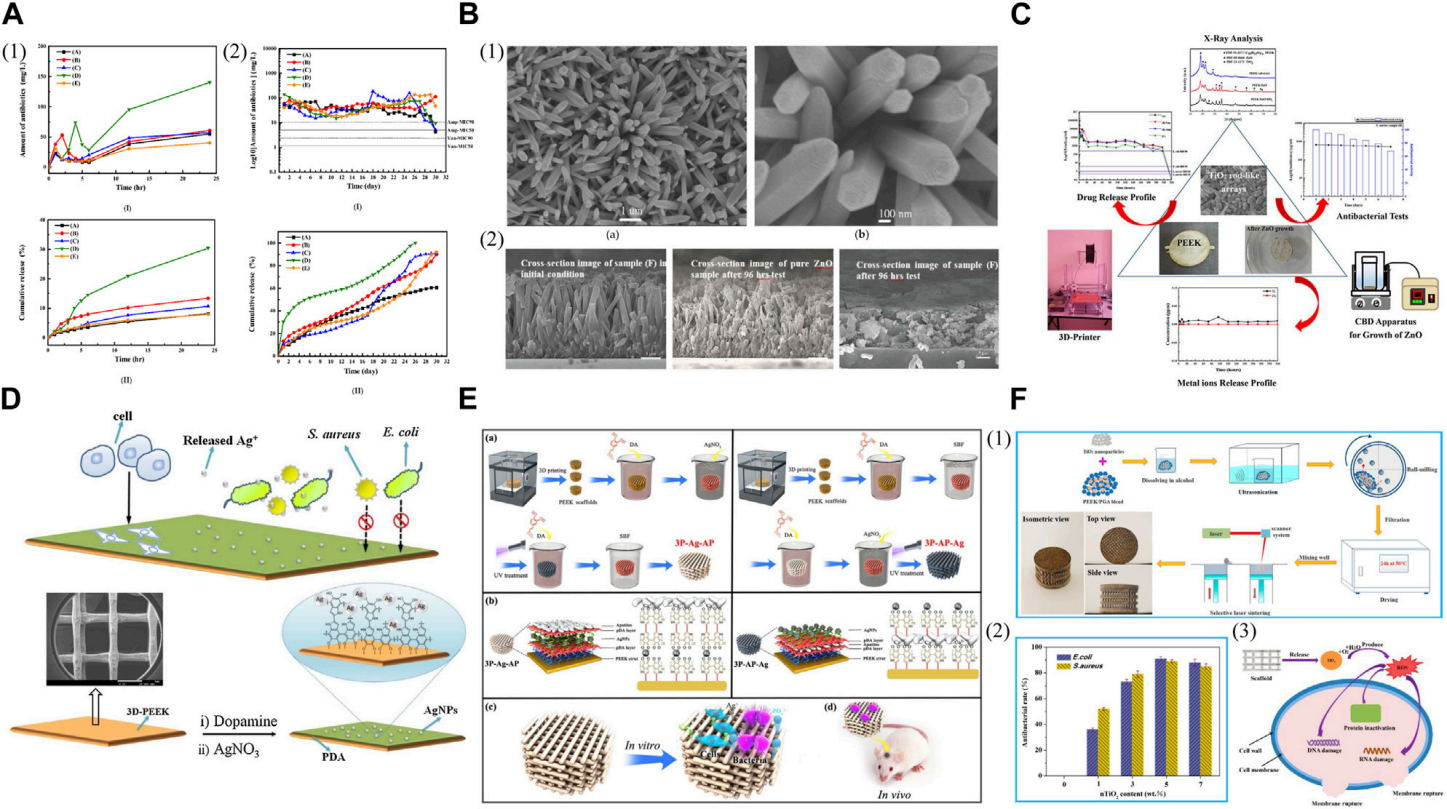
Antibacterial 3D printed PEEK designs. **(A) (1) (I)** Short-term release profiles and **(II)** the cumulative release profiles for the total amount of antibiotic agents released from PEEK into the buffer solution for samples **(A-E)**. **(2) (I)** Long-term release profiles and **(II)** the cumulative release profile for the total amount of antibiotic agents in the buffer solution for samples **(A-E)**. Reproduced with permission (Lau et al., 2020). Copyright 2020, MDPI. **(B) (1)** SEM images of ZnO nanorods/PEEK sample at **(a)** 10 k(X) and **(b)** 50 k(X), respectively. **(2)** SEM images of pure ZnO sample and sample **(F)** before and after drug release testing, respectively. Reproduced with permission (Chen et al., 2019). Copyright 2019, MDPI. **(C)** Antibacterial activity of 3D-printing polyetheretherketone substrates with surface grown TiO_2_/ZnO rodlike arrays. Reproduced with permission (Lau et al., 2022). Copyright 2022, The Authors. published by American Chemical Society. **(D)** Schematic diagram of the preparation of 3D PEEK/Ag scaffold and its biological evaluation. Reproduced with permission (Deng et al., 2017). Copyright 2017, Elsevier B.V. All rights reserved. **(E) (a)** Synthesis of 3P-Ag-AP (left) and 3P-AP-Ag (right) scaffolds with pH-triggered osteopotentiating properties. **(b)** Schematic of the envisioned multilayer architecture of coatings and possible interplay among diverse ingredients of coatings. Schematic of **(c)** *in vitro* and **(d)** *in vivo* tests for the multifunctional scaffolds. Reproduced with permission (Deng et al. (2020)). Copyright 2020, American Chemical Society. **(F) (1)** Schematic of the scaffold preparation process. **(2)** Antibacterial rate of the scaffolds with different nTiO_2_ contents. **(3)** Possible antibacterial mechanisms of PEEK/PGA-nTiO_2_ scaffolds. Reproduced with permission (Shuai et al., 2018). Copyright 2018, MDPI.

The combination of 3D printed PEEK and antimicrobial agents for enhanced antibacterial ability is a commonly used modified method. The application of poly (lactic-co-glycolic acid) (PLGA) polymer as a binder and drug release control component facilitated the coating of ampicillin and/or vancomycin salts on the FDM printed PEEK disks, the antibacterial effects of which lasted for 28 days, and the most effective performance was observed when 50% of each antibiotic agent was loaded (Figure 7A) (Lau et al., 2020). Therefore, this study presents a cost-effective and straightforward technique to prepare PEEK/antibiotic agents/PLGA composite samples. Moreover, antibacterial tests showed that SLS printed porous PEEK/poly (glycolic acid) (PGA) scaffolds loaded with Total Alkaloids from Semen Strychnine (TASS) effectively delivered TASS and exhibited antimicrobial effects against *Escherichia coli (Escherichia coli)* and *Staphylococcus aureus (Staphylococcus aureus) in vitro* (Wu et al., 2020). A chemical synthesis method was employed to cultivate zinc oxide (ZnO) nanorod arrays on FDM printed PEEK substrates as carriers of antibiotics (ampicillin or vancomycin), aiming to provide appropriate antibacterial characteristics to these samples (Figure 7B) (Chen et al., 2019). To avoid the disintegration of ZnO rod-shaped arrays due to the acidic nature of attached antibiotics and to prevent cell cytotoxicity and repaid drug release caused by the release of metal ions, an additional procedure can be conducted to modify the ZnO rod-shaped arrays (Figure 7C) (Lau et al., 2022). The 3D printed PEEK disks were covered with layers of TiO_2_ on the surface of grown ZnO arrays using a chemical bath deposition technique to produce TiO_2_/ZnO/PEEK composites. These composite samples can load various antibiotic agents directly and exhibited the ability to inhibit the growth of 90% (MIC 90) of *E. coli* and *S. aureus,* the antibacterial effects of which remained for nearly 10 days.

Apart from incorporating antibiotics, other agents like silver ions (Ag^+^) and silver nanoparticles (AgNPs) that possess broad-spectrum antibacterial effects are progressively utilized to address the emerging issue of antimicrobial resistance (AMR). The FDM printed PEEK implants were introduced with Ag^+^ using catecholamine chemistry to manage infection of Gram-negative and Gram-positive bacteria while stimulating bone regeneration with improved cell proliferation and increased alkaline phosphatase activity compared to the pure PEEK (Figure 7D) (Deng et al., 2017). Therefore, the potential clinical application of these dual-functional scaffolds for bone tissue repair is significantly expected. Deng et al. (2020) embedded AgNPs onto the first PDA layer and then anchored apatite onto the second PDA layer to create a distinct PDA–Ag–PDA sandwich coating structure, thus imparting pH-responsive ion-releasing behavior upon bacterial activation to the FDM printed porous PEEK scaffolds (Figure 7E). These scaffolds demonstrated remarkable efficacy in eliminating bacteria while displaying satisfactory cytocompatibility and promoting the osteogenic potential of osteoblastic MC3T3-E1 cells. In infected bone defects of critical size, the Ag/apatite co-decorated multifunctional scaffolds also provided excellent bone ingrowth and osseointegration, along with *in vivo* antibacterial effects.

The antibacterial properties of inorganic compounds, like titanium dioxide, have been investigated due to the photocatalytic effects, which occur when titanium dioxide is exposed to UV light, producing reactive oxygen species (ROS) to lyse bacteria. When exposed to bacteria, titanium dioxide also oxidizes coenzyme A within cells, impacting metabolism and creating unfavorable conditions for bacterial survival. Nano titanium dioxide (nTiO₂) was incorporated with SLS printed PEEK/PGA to construct scaffolds with antibacterial activity for bone tissue engineering (Figure 7F) (Shuai et al., 2018). The antibacterial tests towards *E. coli* and *S. aureus* indicated that the scaffolds containing nTiO₂ exhibited potent antibacterial effects attributed to the fact that nTiO₂ caused physical and oxidative harm to bacteria through direct contact and ROS production, leading to the effective destruction of bacterial structure and function.

## 5 Conclusions and outlooks

The anatomical complexity and unique aesthetic characteristics of craniomaxillofacial bones, as well as postoperative infection, pose great clinical challenges for bone reconstruction in this region. Therefore, in order to develop therapeutic strategies with improved efficacy for better craniomaxillofacial bone reconstruction, a thorough understanding of their characteristics and certain design requirements for reparative constructs are reviewed in the first part. Due to the weaknesses of existing methods and the development of AM technology and novel materials, the next part focuses on 3D printed PEEK with the potential to address the pressing need for improved regeneration and functional recovery of bone tissue. Furthermore, specific factors in this region, such as the aesthetic restoration of craniofacial structure and bacterial contamination from the oral cavity, indicate that 3D printed PEEK should have suitable mechanical properties, improved biological activity, and antibacterial capability through a variety of modifications to adapt to bone defects under different causative situations. Therefore, the modified methods for 3D printed PEEK constructs are discussed from the perspective of improved mechanical, biological, and antibacterial performance designs.

Before the widespread application of 3D printed PEEK in the repair of craniomaxillofacial bone defects, several obstacles need to be overcome (Li et al., 2022b; Liu et al., 2022; Zhou et al., 2022; Cheng et al., 2023b; Hu et al., 2023b; Xin et al., 2023). First, there is a contradiction between biological performance and mechanical durability, and sacrificing mechanical strength is sometimes necessary to improve the biological activity of 3D printed PEEK. Moreover, there are concerns about the uncontrolled release of loaded bioactive elements. Combining constructs with versatile and smart hydrogel systems that can sense stimuli in the environment and adapt accordingly may have the potential to provide 3D printed PEEK with a controllable release ability, which may be a solution to this issue worthy of further exploration.

Despite some obstacles, the development of 3D printed PEEK in the repair of craniomaxillofacial bone defects expands the scope of bone reconstruction and holds potential for future clinical use. By leveraging a profound understanding of advanced 3D printing technology, bone regeneration, and the interplay between them, this review may advance the use of 3D printed PEEK constructs as a potent method for more intelligent and personalized bone reconstruction therapy in the craniomaxillofacial region.
